# Correlation Between Spiritual Well-Being and Mental Health Among Older Adults

**DOI:** 10.7759/cureus.91207

**Published:** 2025-08-28

**Authors:** Soumyasree Bose, Sivasankari Varadharasu, Sanjukta Dixit, Reena Singh

**Affiliations:** 1 School of Nursing, Kalinga Institute of Medical Sciences, Bhubaneswar, IND

**Keywords:** elder, mental health, older adults, spirituality and mental health, spiritual well-being

## Abstract

Spiritual well-being and mental health are two essential pillars of a comprehensive healthcare system, particularly in older adults. The present study aimed to determine the correlation between spiritual well-being and mental health among older adults and to identify associations between these variables and selected demographic factors. A correlational study was conducted over nine months among 200 older adults in a selected community area in Bhubaneswar, India. Data were collected using a self-structured sociodemographic proforma, the Warwick-Edinburgh Mental Wellbeing Scale, and the English Spiritual Wellbeing Scale. Interviews were conducted over 15 days to gather information from the study participants. Data analysis was performed using IBM SPSS Statistics for Windows, Version 25.0 (Released 2017; IBM Corp., Armonk, NY, USA). Spearman’s correlation was applied to examine the relationship between spiritual well-being and mental health. The chi-square test, Fisher’s exact test, t test, and ANOVA were used to analyze associations with sociodemographic variables. A p-value < 0.05 was considered statistically significant. The study found no correlation between spiritual well-being and mental health. Similarly, no significant association was observed between sociodemographic variables and spiritual well-being. However, age (p = 0.048), education (p = 0.002), and socioeconomic status (p = 0.005) were significantly correlated with mental health. The findings indicated that a greater proportion of older adults fell into the categories of partial and poor spiritual well-being compared to complete spiritual well-being. Most participants were categorized as having fair mental well-being. Overall, no correlation was observed between spiritual well-being and mental health among older adults.

## Introduction

The term “geriatric” refers to older individuals or the care of older adults. According to the World Health Organization, the proportion of individuals over 60 years is projected to increase from 12% to 22% between 2015 and 2050. By 2050, 80% of older adults will live in low- or middle-income countries. The global population is aging much more rapidly than in previous decades. Since 2020, the number of people over the age of 60 has exceeded the number of children under five years old [1]. Older adults with mental illness often present more physical than psychological symptoms, making diagnosis and treatment difficult. Many are likely unaware of their psychological problems. Numerous observational studies have linked spirituality to geriatric care, demonstrating positive health effects among older adults [2].

The word “spirituality” originates from the Latin term “spiritus,” meaning “the breath of life.” Spirituality holds different meanings for different individuals. The Cambridge Dictionary defines spirituality as “the quality that involves deep feelings and beliefs of a religious nature, rather than the physical parts of life” [3]. Spiritual well-being refers to a state that enables individuals to understand the meaning of life and live purposefully with the guidance of a transcendent source. Although religiosity is more common among older adults compared to simple spiritual practices, the two concepts differ. Religiosity refers to the specific practices of a particular religion in which an individual has faith, whereas spirituality is a broader concept that is not necessarily tied to a specific belief or faith. Nevertheless, spirituality is often intertwined with religiosity.

Mental health is a state of well-being that enables individuals to cope with stress, develop their abilities, and contribute to society. Good mental health involves more than just the absence of mental illness [4]; it reflects a condition in which a person feels fulfilled and functions effectively in social settings.

Spirituality and religion have been shown to improve both physical and mental health, thereby reducing the risk of certain diseases such as heart disease, emphysema, stroke, renal failure, and others [5]. They also influence overall mortality among older adults. Spirituality and religion are positively correlated with health, cognitive function, and stable marriages, and negatively correlated with suicide, misconduct, criminal activity, and substance use [2].

Maintaining good spiritual health may or may not directly affect mental health, and this relationship requires further investigation. At the same time, awareness of psychological problems among older adults is crucial. Given the growing proportion of older adults, about 9.5% of the population in Odisha, India [6], this study was necessary. It is essential to understand the prevalence of psychological illness among older adults and explore effective interventions. Cost-effective treatment modalities with a scientific basis are needed. As spirituality is more commonly practiced among older adults, assessing its correlation with mental health is particularly important.

## Materials and methods

Design of the study

The present study is a quantitative, nonexperimental, descriptive, cross-sectional study conducted in the Niladri Vihar community area of Bhubaneswar, Odisha, India. The aim was to assess the correlation between spiritual well-being and mental health. Niladri Vihar was deliberately chosen, as approximately 9-10% of its population are older adults. The study was conducted over nine months using a cluster sampling technique. Older adults aged 65-85 years were selected from the streets of Sitanath Nagar, Rickshaw Colony, and Adarsh Basti, among other areas of Niladri Vihar. A list of eligible older adults was obtained from the local corporator. One-stage cluster sampling was employed, and all older adults who met the inclusion criteria were interviewed, with data subsequently collected.

The research variables included spiritual well-being, mental health, and demographic characteristics such as age, gender, religion, education, marital status, employment status, monthly family income, spiritual practices, number of hours per day spent on spiritual practices, mode of spiritual practice, family history of mental illness, history of medical illness, current living arrangements, means of meeting expenses, and fear of death.

The sample size was calculated using the standard formula for descriptive studies:

\[n = \frac{\text{deff} \times N p (1-p)}{\dfrac{d^{2}}{z^{2}_{1-\alpha/2}} (N-1) + p(1-p),}\]

where the population size (N) was 10,000 (for finite population correction factor), the hypothesized frequency of outcome factor in the population (p) was 9.5% ± 5, the confidence limit (d) was 5%, and the design effect for cluster survey (deff) was 1.5. At the 95% confidence level, the required sample size was 199, which was approximated to 200. Considering that 9.5% of Odisha’s population are older adults (Odisha Economic Survey, 2020-21), with a 5% confidence limit and 1.5 design effect, 200 participants were selected.

The 200 older adults were selected from the streets of Sitanath Nagar, Rickshaw Colony, and Adarsh Basti, among other areas of Niladri Vihar in Bhubaneswar, Odisha, India. The inclusion criteria consisted of individuals aged 65-85 years who understood Odia or English and provided written consent to participate in the study. Older adults who did not have faith in spirituality, were diagnosed with mental disorders such as psychosis or organic brain disorders, or were in an extremely physically debilitated condition were excluded, as data collection from these individuals would have been both difficult and unreliable. Those engaged in other practices such as music therapy, relaxation techniques, or yoga were also excluded, as these could interfere with the assessment of spiritual well-being and mental health.

The purpose of the study was explained to participants, and signed informed consent was obtained. A 15-item self-structured sociodemographic proforma was used to assess variables including age, gender, religion, educational background, marital status, employment status, monthly family income, spiritual practice, number of hours per day spent on spiritual activities, nature of spiritual practice, family history of mental illness, history of medical illness, current living arrangements, source of current expenses, and fear of death. The proforma was prepared by the authors and validated by seven experts, including two psychiatrists, two clinical psychologists, two mental health nurses, and one biostatistician. A pilot study was conducted in May prior to the main study. The tool was developed solely for academic purposes and was not used for any economic or commercial application; it may also have different interpretations in other contexts.

To assess mental health, 14 items of the Warwick-Edinburgh Mental Wellbeing Scale (WEMWBS) were used, while spiritual well-being was measured using 10 items of the Spiritual Wellbeing Scale (SWBS).

Study tool

Demographic and Research Variables

Demographic and research variables were collected from older adults in the Niladri Vihar community area using the interview method. These variables were scored and used solely for analysis purposes.

WEMWBS

The WEMWBS is a 14-item questionnaire designed to evaluate the mental health of the study participants. The total score ranges from 14 to 70, with higher scores indicating better mental well-being. According to the total score, mental health status was categorized as follows: a score between 56 and 70 indicated complete mental well-being, 42 to 55 indicated fair mental well-being, 28 to 41 indicated partial mental well-being, and 14 to 27 indicated poor mental well-being.

SWBS

The SWBS is a 10-item questionnaire used in this study to assess the perceived spiritual quality of life. Scores were obtained by summing the ratings across items, producing a possible range from 10 to 50. According to the total score, spiritual well-being was classified as follows: scores between 37 and 49 indicated complete spiritual well-being, 24 to 36 indicated partial spiritual well-being, and 11 to 23 indicated poor spiritual well-being.

Data description and analysis

Descriptive and inferential statistical analyses were conducted using IBM SPSS Statistics for Windows, Version 25.0 (Released 2017; IBM Corp., Armonk, NY, USA) and Microsoft Excel version 16 (Microsoft Corporation, Redmond, WA, USA). Data were presented as numbers, percentages, means, and standard deviations for continuous and categorical variables. The correlation between spiritual well-being and mental health was assessed using Spearman’s correlation. Associations between demographic factors, spiritual well-being, and mental health were analyzed using chi-square tests, Fisher’s exact test, t tests, and ANOVA. For the study, statistical significance was set at p < 0.05.

## Results

A total of 200 participants who met the inclusion and exclusion criteria were included in the analysis. Frequencies and percentages were calculated to evaluate the levels of spiritual well-being and mental health (Table 1) as well as the sociodemographic variables (Table 2). Spearman’s correlation test was used to examine the relationship between spiritual well-being and mental well-being. Chi-square tests, Fisher’s exact test, linear regression, t-test, and ANOVA were performed to assess associations between spiritual well-being, mental health, and selected sociodemographic variables.

**Table 1:** Description of spiritual well-being and mental health of the samples using frequency and percentage

Level of spiritual well-being and mental health		Frequency	Percentage
Spiritual well-being	Complete spiritual well-being	0	0.0%
	Partial spiritual well-being	174	87.0%
	Poor spiritual well-being	26	13.0%
Mental health	Complete mental well-being	43	21.5%
	Fair mental well-being	155	77.5%
	Partial mental well-being	2	1.0%
	Poor mental well-being	0	0.0%

**Table 2:** Frequency and percentage distribution of participants by sociodemographic variables

Variables		Frequency	Percentage
Gender	Male	96	48.0%
	Female	104	52.0%
	Others	0	0.0%
Religion	Hindu	172	86.0%
	Muslim	9	4.5%
	Christian	19	9.5%
	Others	0	0.0%
Education	No formal education	38	19.0%
	Primary education	59	29.5%
	Secondary education	50	25.0%
	Higher secondary	24	12.0%
	Graduation	29	14.5%
	Post-graduation and above	0	00.0%
Marital status	Unmarried	0	00.0%
	Married	133	66.5%
	Divorcee	0	00.0%
	Widow/widower	67	33.5%
Employment status	Employed	5	2.5%
	Unemployed	131	65.5%
	Self-employed	20	10.0%
	Retired	44	22.0%
Monthly income	≤9226	3	1.5%
	9232-27,648	108	54.0%
	27,654-46,089	67	33.5%
	46,095-68,961	22	11.0%
	68,967-92,185	0	00.0%
	92,191-184,370	0	00.0%
	≥184,376	0	00.0%
Spiritual practice	Yes	165	82.5%
	No	0	00.0%
	Sometimes	35	17.5%
Hours of spiritual practice	Within one hour	182	91.0%
	One to two hours	18	9.0%
	More than two hours	0	00.0%
Mode of practice	Chanting	67	33.5%
	Prayer	21	10.5%
	Bhajana	0	00.0%
	Puja	112	56.0%
	Others, specify	0	00.0%
Family history of mental illness	Yes, specify	11	5.5%
	No	189	94.5%
Medical illness	Yes, specify	95	47.5%
	No	105	52.5%
Presently staying with	Spouse only	25	12.5%
	Spouse and children	51	25.5%
	Spouse, children, and grandchildren	57	28.5%
	Children only	12	6.0%
	Children and grandchildren	55	27.5%
Current way of meeting daily expenses	Pension	35	17.5%
	Interest in other sources	2	1.0%
	Children	116	58.0%
	Other	47	23.5%
Fear of death	Yes	119	59.5%
	No	81	40.5%

With respect to spiritual well-being, the majority of participants, 174 (87.0%), demonstrated partial spiritual well-being, while 26 (13.0%) exhibited poor spiritual well-being. None of the participants demonstrated complete spiritual well-being. With respect to mental health, most participants, 155 (77.5%), demonstrated fair mental well-being, while 43 (21.5%) demonstrated complete mental well-being. Partial mental well-being was observed in two (1.0%) participants, and none of the participants demonstrated poor mental well-being.

The gender of the study participants included 104 (52.0%) females, forming the majority, while the remaining 96 (48.0%) were males. With regard to religion, Hindu participants were the largest group, comprising 172 (86.0%), followed by 19 (9.5%) Christians and nine (4.5%) Muslims. No participants from other religions were reported. In terms of education, 59 (29.5%) of the participants had primary education, 50 (25.0%) had secondary education, and 38 (19.0%) had no formal education. Additionally, 29 (14.5%) participants had completed graduation, and 24 (12.0%) had completed higher secondary education, while none had attained post-graduation or higher. Regarding marital status, 133 (66.5%) participants were married and 67 (33.5%) were widows/widowers, with no unmarried or divorced participants. For employment status, the majority, 131 (65.5%), were unemployed, followed by 44 (22.0%) retired participants, 20 (10.0%) who were self-employed, and five (2.5%) who were employed.

Family monthly income showed that the majority, 108 (54.0%), had an income of 9,232-27,648 Rs. A total of 67 (33.5%) had a monthly income of 27,654-46,089 Rs., and 22 (11.0%) had an income of 46,095-68,961 Rs. Only 3 (1.5%) had an income of ≤9226 Rs., while no participants reported incomes of 68,967-92,185 Rs., 92,191-184,370 Rs., or ≥184,376 Rs. With regard to spiritual practice, 165 (82.5%) participants reported practicing spirituality regularly, while 35 (17.5%) practiced it only some of the time. The majority, 182 (91.0%), practiced spirituality for less than one hour daily, while 18 (9.0%) practiced for one to two hours, and none practiced for more than two hours. In terms of mode of practice, 112 (56.0%) engaged in Puja, 67 (33.5%) in chanting, and 21 (10.5%) in prayer.

Concerning health background, 189 (94.5%) participants reported no family history of mental illness, while 11 (5.5%) did. Regarding medical illness, 105 (52.5%) had no medical illness, while 95 (47.5%) reported having one. In terms of living arrangements, 57 (28.5%) participants lived with their spouse, children, and grandchildren, 55 (27.5%) lived with children and grandchildren only, and 51 (25.5%) lived with a spouse only. For meeting daily expenses, 116 (58.0%) depended on their children, 47 (23.5%) relied on other sources, 35 (17.5%) relied on pensions, and two (1.0%) used interest from other sources. Finally, 119 (59.5%) participants reported a fear of death, while 81 (40.5%) did not.

The sociodemographic variables, gender, marital status, spiritual practice, duration of spiritual practice, mode of spiritual practice, family history of mental illness, medical history, current living arrangement, and fear of death, showed no significant association with spiritual well-being (Table 3).

**Table 3:** Association between spiritual well-being and sociodemographic variables of study participants using the chi-square test SWBS, Spiritual Wellbeing Scale

Demographic variable	Frequency and percentage of SWBS						Chi-square	Level of freedom	p-Value
	Complete		Partial		Poor				
	N	%	N	%	N	%			
Gender									
Male	0	0.00%	86	89.6%	10	10.4%	1.089	1	0.297
Female	0	0.00%	88	84.60%	16	15.40%			
Others	0	0.00%	0	0.00%	0	0.00%			
Marital status									
Unmarried	0	0.00%	0	0.00%	0	0.00%	0.58	1	0.446
Married	0	0.00%	114	85.70%	19	14.30%			
Divorcee	0	0.00%	0	0.00%	0	0			
Widow/widower	0	0.00%	60	89.60%	7	10.40%			
Spiritual practice									
Yes	0	0.00%	146	88.50%	19	11.50%	1.838	1	0.175
No	0	0.00%	0	0.00%	0	0.00%			
Sometimes	0	0.00%	28	80.00%	7	20.00%			
Hours of spiritual practice									
Within one hour	0	0.00%	158	86.80%	24	13.20%	0.062	1	0.803
One to two hours	0	0.00%	16	88.90%	2	11.10%			
More than two hours	0	0.00%	0	0.00%	0	0.00%			
Mode of spiritual practice									
Chanting	0	0.00%	57	85.10%	10	14.90%	0.469	2	0.791
Prayer	0	0.00%	19	90.50%	2	9.50%			
Bhajana	0	0.00%	0	0.00%	0	0.00%			
Puja	0	0.00%	98	87.50%	14	12.50%			
Others, specify	0	0.00%	0	0.00%	0	0.00%			
Family history of mental illness									
Yes, specify	0	0.00%	10	90.90%	1	9.10%	0.157	1	0.692
No	0	0.00%	164	86.80%	25	13.20%			
Medical illness									
Yes, specify	0	0.00%	83	87.40%	12	12.60%	0.022	1	0.883
No	0	0.00%	91	86.70%	14	13.30%			
Presently staying with									
Spouse only	0	0.00%	19	76.00%	6	24.00%	6.443	4	0.168
Spouse and children	0	0.00%	48	94.10%	3	5.90%			
Spouse, children, and grandchildren	0	0.00%	47	82.50%	10	17.50%			
Children only	0	0.00%	11	91.70%	1	8.30%			
Children and grandchildren	0	0.00%	49	89.10%	6	10.90%			
Fear of death									
Yes	0	0.00%	104	87.40%	15	12.60%	0.041	1	0.84
No	0	0.00%	70	86.40%	11	13.60%			

The sociodemographic variables, religion, education, employment, socioeconomic status, and current means of meeting daily expenses, showed no significant association with spiritual well-being (Table 4).

**Table 4:** Association between spiritual well-being and sociodemographic variables of study participants using Fisher’s exact test SWBS, Spiritual Wellbeing Scale

Demographic variable	Frequency and percentage of SWBS						Fisher’s exact test	p-Value
Religion								
Hindu	0	0.00%	150	87.20%	22	12.80%	1.092	0.575
Muslim	0	0.00%	7	77.80%	2	22.20%		
Christian	0	0.00%	17	89.50%	2	10.50%		
Others, specify	0	0.00%	0	0	0	0		
Education								
No formal education	0	0.00%	34	89.50%	4	10.50%	1.453	0.848
Primary	0	0.00%	52	88.10%	7	11.90%		
Secondary	0	0.00%	41	82.00%	9	18.00%		
Higher secondary	0	0.00%	21	87.50%	3	12.50%		
Graduation	0	0.00%	26	89.70%	3	10.30%		
Post-graduation and above	0	0.00%	0	0	0	0		
Employment status								
Employed	0	0.00%	5	100.00%	0	0.00%	0.656	0.901
Unemployed	0	0.00%	114	87.00%	17	13.00%		
Self-employed	0	0.00%	18	90.00%	2	10.00%		
Retired	0	0.00%	37	84.10%	7	15.90%		
Monthly family income								
≤9226	0	0.00%	3	100.00%	0	0.00%	0.523	0.912
9232-27,648	0	0.00%	94	87.00%	14	13.00%		
27,654-46,089	0	0.00%	57	85.10%	10	14.90%		
46,095-68,961	0	0.00%	20	90.90%	2	9.10%		
68,967-92,185	0	0.00%	0	0.00%	0	0.00%		
92,191-184,370	0	0.00%	0	0.00%	0	0.00%		
≥184,376	0	0.00%	0	0.00%	0	0.00%		
Current way of meeting daily expenses								
Pension	0	0.00%	29	82.90%	6	17.10%	1	0.777
Interest in other sources	0	0.00%	2	100.00%	0	0.00%		
Children	0	0.00%	102	87.90%	14	12.10%		
Other	0	0.00%	41	87.20%	6	12.80%		

Table 5 shows a significant association between the sociodemographic variables education (Fisher’s exact = 20.167, p = 0.002) and monthly family income (Fisher’s exact = 16.740, p = 0.005) with the mental health of the study participants.

**Table 5:** Association between mental health and sociodemographic variables of participants using Fisher’s exact test Education and monthly family income are statistically significant, as indicated by asterisks (^*^). WEMWBS, Warwick-Edinburgh Mental Wellbeing Scale

Demographic variable	Frequency and percentage of WEMWBS								Fisher’s exact test	p-Value
	Complete		Fair		Partial		Poor			
	N	%	N	%	N	%	N	%		
Gender										
Male	23	24.00%	72	75.00%	1	1.00%	0	0.00%	0.92	0.745
Female	20	19.20%	83	79.80%	1	1.00%	0	0.00%		
Others	0	0.00%	0	0.00%	0	0.00%	0	0.00%		
Religion										
Hindu	41	23.80%	129	75.00%	2	1.20%	0	0.00%	5.106	0.296
Muslim	0	0.00%	9	100.00%	0	0.00%	0	0.00%		
Christian	2	10.50%	17	89.50%	0	0.00%	0	0.00%		
Others, specify	0	0.00%	0	0	0	0	0	0.00%		
Education^*^										
No formal education	8	21.10%	30	78.90%	0	0.00%	0	0.00%	20.167	0.002
Primary	21	35.60%	38	64.40%	0	0.00%	0	0.00%		
Secondary	6	12.00%	44	88.00%	0	0.00%	0	0.00%		
Higher secondary	6	25.00%	16	66.70%	2	8.30%	0	0.00%		
Graduation	2	6.90%	27	93.10%	0	0.00%	0	0.00%		
Post-graduation and above	0	0.00%	0	0	0	0	0	0.00%		
Marital status										
Unmarried	0	0.00%	0	0.00%	0	0.00%	0	0.00%	0.915	0.689
Married	30	22.60%	101	75.90%	2	1.50%	0	0.00%		
Divorcee	0	0.00%	0	0.00%	0	0	0	0.00%		
Widow/widower	13	19.40%	54	80.60%	0	0.00%	0	0.00%		
Employment status										
Employed	3	60.00%	2	40.00%	0	0.00%	0	0.00%	10.248	0.1
Unemployed	29	22.10%	101	77.10%	1	0.80%	0	0.00%		
Self-employed	5	25.00%	14	70.00%	1	5.00%	0	0.00%		
Retired	6	13.60%	38	86.40%	0	0.00%	0	0.00%		
Monthly family income^*^										
≤9226	3	100.00%	0	0.00%	0	0.00%	0	0.00%	16.740	0.005
9232-27,648	29	26.90%	78	72.20%	1	0.90%	0	0.00%		
27,654-46,089	8	11.90%	58	86.60%	1	1.50%	0	0.00%		
46,095-68,961	3	13.60%	19	90.90%	0	0.00%	0	0.00%		
68,967-92,185	0	0.00%	0	0.00%	0	0.00%	0	0.00%		
92,191-184,370	0	0.00%	0	0.00%	0	0.00%	0	0.00%		
≥184,376	0	0.00%	0	0.00%	0	0.00%	0	0.00%		
Spiritual practice										
Yes	40	24.20%	123	74.50%	2	1.20%	0	0.00%	4.659	0.077
No	0	0.00%	0	0.00%	0	0.00%	0	0.00%		
Sometimes	3	8.60%	32	91.40%	0	0.00%	0	0.00%		
Hours of spiritual practice										
Within one hour	40	22.00%	140	76.90%	2	1.10%	0	0.00%	0.555	0.808
One to two hours	3	16.70%	15	83.30%	0	0.00%	0	0.00%		
More than two hours	0	0.00%	0	0.00%	0	0.00%	0	0.00%		
Mode of spiritual practice										
Chanting	12	17.90%	55	82.10%	0	0.00%	0	0.00%	4.767	0.286
Prayer	2	9.50%	19	90.50%	0	0.00%	0	0.00%		
Bhajana	0	0.00%	0	0.00%	0	0.00%	0	0.00%		
Puja	29	25.90%	81	72.30%	2	1.80%	0	0.00%		
Others, specify	0	0.00%	0	0.00%	0	0.00%	0	0.00%		
Medical illness										
Yes, specify	23	24.20%	70	73.70%	2	2.10%	0	0.00%	2.797	0.212
No	20	19.00%	85	81.00%	0	0.00%	0	0.00%		
Presently staying with										
Spouse only	2	8.00%	23	92.00%	0	0.00%	0	0.00%	9.445	0.225
Spouse and children	15	29.40%	35	68.60%	1	2.00%	0	0.00%		
Spouse, children, and grandchildren	13	22.80%	43	75.40%	1	1.80%	0	0.00%		
Children only	4	33.30%	8	66.70%	0	0.00%	0	0.00%		
Children and grandchildren	9	16.40%	46	83.60%	0	0.00%	0	0.00%		
Current way of meeting daily expenses										
Pension	5	14.30%	30	85.70%	0	0.00%	0	0.00%	11.462	0.068
Interest in other sources	0	0.00%	2	100.00%	0	0.00%	0	0.00%		
Children	23	19.80%	93	80.20%	0	0.00%	0	0.00%		
Other	15	31.90%	30	63.80%	2	4.30%	0	0.00%		
Fear of death										
Yes	26	21.80%	91	76.50%	2	1.70%	0	0.00%	1.037	0.653
No	17	21.00%	64	79.00%	0	0.00%	0	0.00%		

Table 6 shows the level of association between participants’ age and partial or poor spiritual well-being, indicating a significant association with age.

**Table 6:** Unpaired t test analysis of participant age in relation to partial and poor spiritual well-being SWBS, Spiritual Wellbeing Scale

SWBS	N	Mean	SD	Unpaired t test	p-Value
Partial spiritual well-being	174	71.41	5.469	-1.434	0.153
Poor spiritual well-being	26	73.12	6.719	-1.232	0.228

Table 7 shows the level of association between participants’ age and complete, fair, and partial mental well-being, indicating a significant association (F = 3.075, p = 0.048).

**Table 7:** One-way ANOVA analysis of participant age across complete, fair, and partial mental well-being ^*^ Statistically significant WEMWBS, Warwick-Edinburgh Mental Wellbeing Scale

WEMWBS	N	Mean	SD	One-way ANOVA	p-Value
Complete mental well-being	43	70.09	5.656	3.075	0.048^*^
Fair mental well-being	155	72.13	5.599		
Partial mental well-being	2	66.5	2.121		

**Figure 1:**
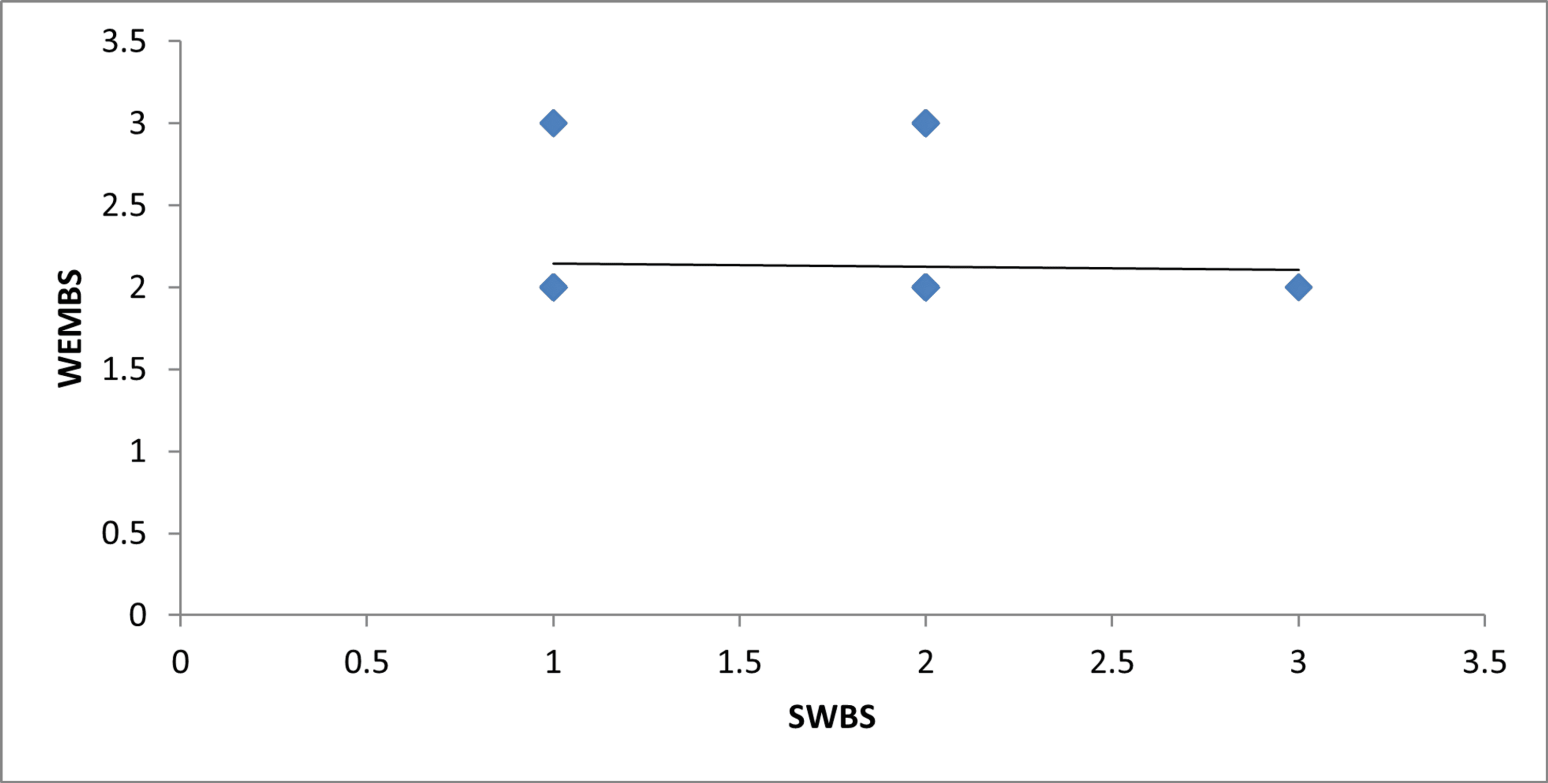
Correlation between spiritual well-being and mental health among the study participants The figure shows that there is no correlation between spiritual well-being and mental health among the study participants.

The correlation coefficient (ρ = 0.107, p = 0.133) was not statistically significant at p < 0.05, indicating no correlation between spiritual well-being and mental health among the study participants (Figure 1).

## Discussion

The present analysis shows that among the older adults of the selected community area, the majority (87.0%) exhibited partial spiritual well-being, followed by poor spiritual well-being. No participants demonstrated complete spiritual well-being. Regarding mental health, most participants (77.5%) had fair mental well-being, followed by complete mental well-being, and only 1% exhibited partial mental well-being. None of the participants had poor mental well-being.

The results also indicate no correlation between spiritual well-being and mental health (ρ = 0.107, p = 0.133), which contrasts with the findings of Aydın et al. (2020), who observed a negative correlation between overall spiritual well-being and mental illnesses such as depression, anxiety, negative self-perception, and somatization [7]. This suggests that regular spiritual practice may not directly impact the mental health of older adults.

No significant associations were observed between spiritual well-being and age, gender, religion, education, marital status, employment, socioeconomic status, spiritual practice, hours of spiritual practice, mode of spiritual practice, family history of mental illness, medical history, current living arrangements, way of meeting daily expenses, or fear of death.

Mental health, however, showed significant associations with age (p = 0.048), which aligns with WHO observations that disorders such as depression and anxiety are common among older adults [8]. Education was also significantly associated with mental health (p = 0.002), consistent with findings from Sperandei et al. (2021), which reported that lower educational attainment is linked to poorer mental health outcomes and increased psychological distress [9].

Socioeconomic status was another significant factor (p = 0.005), supporting observations by Sánchez-Moreno et al. (2024), who noted that older adults with limited income or financial difficulties are more prone to depression, anxiety, and social isolation, which can also affect physical health [10]. Similarly, Belo et al. (2020) reported that higher education contributes to better psychological adjustment among older adults [11], and Zhou et al. (2021) found that higher socioeconomic status is associated with lower prevalence of mental health disorders such as depression [12].

The remaining sociodemographic variables, gender, religion, employment, spiritual practice, hours of spiritual practice, mode of spiritual practice, family history of mental illness, medical history, living arrangements, way of meeting daily expenses, and fear of death, showed no significant associations with mental health. Given the lack of significant associations for these variables, the null hypothesis can be accepted for all cases related to age, gender, religion, education, marital status, family type, monthly family income, institution, year of study, branch, schooling board, and speaking courses.

Limitations

This study had certain limitations, including the extensive time required for data collection and the focus on a single community area, which may limit generalizability. Additionally, spiritual well-being might be more accurately assessed through a qualitative approach.

## Conclusions

Partial spiritual well-being was the most common among the study participants, with no participants achieving complete spiritual well-being. Most participants exhibited fair mental well-being, and none had poor mental well-being. No correlation was found between spiritual well-being and mental health, indicating that spiritual practices alone may not influence mental health. Age, education, and socioeconomic status were significantly associated with mental health. Future studies are recommended to include larger sample sizes, consider cultural differences, and assess spiritual well-being using qualitative methods for a more comprehensive understanding.

## Appendices

**Table 8:** Spiritual Wellbeing Scale The following instructions were given to the participants: Please mark the answer that best applies to you: strongly agree, agree, neither agree nor disagree, disagree, or strongly disagree. The work “Higher Power” or “God” refers to God, Buddha, or any other Supreme Being/Higher Power/Life Force you may believe in. Please circle only one answer per line.

	Strongly agree	Agree	Neither agree nor disagree	Disagree	Strongly disagree
I don’t know who I am, where I come from, or where I am going.	1	2	3	4	5
I believe that God/a Higher Power loves me and cares about me.	1	2	3	4	5
I have a personally meaningful relationship with God/a Higher Power.	1	2	3	4	5
I feel very fulfilled and satisfied with my life.	1	2	3	4	5
I don’t get much personal strength and support from God/a Higher Power.	1	2	3	4	5
I believe that God/a Higher Power is concerned about my problems.	1	2	3	4	5
I feel good about my future.	1	2	3	4	5
My life doesn’t have much meaning.	1	2	3	4	5
My relationship with God/a Higher Power contributes to my sense of well-being.	1	2	3	4	5
10. I believe there is some real purpose for my life.	1	2	3	4	5

**Table 9:** Warwick-Edinburgh Mental Wellbeing Scale The following instructions were given to the participants: Below are some statements about feelings and thoughts. Please tick the box that best describes your experience of each over the last two weeks.

	None of the time	Rarely	Some of the time	Often	All of the time
1. I’ve been feeling optimistic about the future	1	2	3	4	5
2. I’ve been feeling useful	1	2	3	4	5
3. I’ve been feeling relaxed	1	2	3	4	5
4. I’ve been feeling interested in other people	1	2	3	4	5
5. I’ve had energy to spare	1	2	3	4	5
6. I’ve been dealing with problems well	1	2	3	4	5
7. I’ve been thinking clearly	1	2	3	4	5
8. I’ve been feeling good about myself	1	2	3	4	5
9. I’ve been feeling close to other people	1	2	3	4	5
10. I’ve been feeling confident	1	2	3	4	5
11. I’ve been able to make up my own mind about things.	1	2	3	4	5
12. I’ve been feeling loved	1	2	3	4	5
13. I’ve been interested in new things	1	2	3	4	5
14. I’ve been feeling cheerful	1	2	3	4	5
